# Effects of oral intake of water in patients with oropharyngeal dysphagia

**DOI:** 10.1186/1471-2318-11-9

**Published:** 2011-03-01

**Authors:** Martha JP Karagiannis, Leonie Chivers, Tom C Karagiannis

**Affiliations:** 1 West Wimmera Health Service, Nhill, Victoria, Australia; 2 Darebin Aged Care Health Service, Melbourne, Victoria, Australia; 3 Loddon Mallee Region, Victorian Infant Hearing Screening Program, Royal Children's Hospital, Melbourne, Victoria, Australia; 4 Epigenomic Medicine, BakerIDI Heart and Diabetes Institute, The Alfred Medical Research and Education Precinct, Melbourne, Victoria, Australia; 5 Department of Pathology, The University of Melbourne, Parkville, Victoria, Australia

## Abstract

**Background:**

Dysphagia is associated with numerous medical conditions and the major intervention to avoid aspiration in people with dysphagia involves modifying the diet to thickened fluids. This is associated with issues related to patient quality of life and in many cases non-compliance leading to dehydration. Given these concerns and in the absence of conclusive scientific evidence, we designed a study, to further investigate the effects of oral intake of water in people with dysphagia.

**Methods:**

We monitored lung related complications, hydration levels and assessed quality of life in two groups of people with dysphagia. The control group was allowed only thickened fluids and patients in the intervention group were allowed access to water for a period of five days.

**Results:**

Our findings indicate a significantly increased risk in the development lung complications in patients given access to water (6/42; 14.3%) compared to the control group (0/34; no cases). We have further defined patients at highest risk, namely those with degenerative neurologic dysfunction who are immobile or have low mobility. Our results indicate increased total fluid intake in the patients allowed access to water, and the quality of life surveys, albeit from a limited number of patients (24% of patients), suggest the dissatisfaction of patients to diets composed of only thickened fluids.

**Conclusions:**

On the basis of these findings we recommend that acute patients, patients with severe neurological dysfunction and immobility should be strongly encouraged to adhere to a thickened fluid or modified solid consistency diet. We recommend that subacute patients with relatively good mobility should have choice after being well-informed of the relative risk.

**Trial registration:**

Australia and New Zealand Clinical Trials Register (ANZCTR): ACTRN12608000107325

## Background

Oropharyngeal dysphagia is a common clinical problem, predominantly in the elderly, and is associated with numerous pathologies including, cerebrovascular accident (CVA; stroke), neurodegenerative diseases (e. g. Alzheimer's disease, Parkinson's disease and multiple sclerosis), certain advanced cancers, particularly head and neck, and may result from a traumatic injury [1-6]. Dysphagia has been most widely investigated in the context of CVA where the incidence of swallowing difficulties ranges from 40 to 70% of patients [7-9]. A major complication of dysphagia is aspiration which results in morbidity due to the development of pneumonia - referred to as aspiration pneumonia [8,10-13]. The facts that aspiration occurs in about 40-50% of CVA patients and the mortality from aspiration pneumonia has been reported to be as high as 6% in the first year after a CVA, highlight the extent of the problem [11-18]. The causal relationship between aspiration and the development of pneumonia is well-established [19-22].

Likewise, it is well known that thin liquid is the most likely consistency to be aspirated [23]. The conventional treatment options that are recommended for patients that aspirate thin liquids are limited. Although postural interventions have been shown to be beneficial, the orthodox treatment remains consumption of thickened liquids, typically to an extremely, moderately or mildly thick consistency and a diet consisting of modified solids [24-26]. However, there are major concerns associated with the prescription of thickened fluids and modified solid consistencies [27,28]. These are predominantly patient dissatisfaction and in certain cases non-compliance leading to medical problems associated with hydration and malnutrition [27,28].

The problems associated with patient disapproval of thickened liquids and modified solid consistency diets are well known. They have created an enduring debate related to the management of people with dysphagia who aspirate on thin liquids. In an attempt to overcome these problems an authoritative protocol was developed at the Frazier Rehab Center in Louisville, US about 25 years ago for the management of patients with dysphagia. This free water protocol provides detailed guidelines and strict procedures for allowing access of water to people with dysphagia who, have insurance coverage as well as the ability to tolerate three hours of physical rehabilitation daily for six days per week. Although the protocol is based on a solid rationale for the provision of water and is supported by scientific research, albeit limited, there is still much confusion in the clinic.

An important study published in 1997 addressed the issues related to the effects of water on aspiration pneumonia, hydration and quality of life in patients known to aspirate thin liquids [29]. This one year randomised-control study compared two groups of stroke patients who were known to aspirate on thin fluids [29]. The control group of ten had only thickened fluids and the study group of ten had thickened fluids as well as access to free water[29]. The findings indicated that there were no instances of pneumonia, dehydration or complications in either group[29]. However patient satisfaction was much superior in the study group whereas only one person in the control group was happy with the thickened fluids [29].

However, the limitation of this study is that it only involved 20 very carefully selected patients. Namely, only patients in a stroke rehabilitation unit or who had suffered a CVA within three weeks of admission were included in the study [29]. Patients with previous CVA, neurodegenerative diseases, multiple medical diagnoses and those unable to self-feed were excluded from the study. Due to the limitations of the study, even though there were no instances of pneumonia, dehydration or other complications, the major conclusion drawn was that access to water should only be provided to patients refusing thickened liquids or when hydration becomes an issue of concern [29].

Given that modified diets raise serious clinical management problems with respect to hydration and quality of life and in the absence of conclusive scientific evidence, numerous hospitals, medical centres and aged care homes have adopted policies allowing free access to water. However, this is not a uniform policy with the argument being that it is predominantly based on anecdotal support, since there is minimal documented evidence to reflect the practise. Therefore, we designed a randomised-control prospective study, to further investigate the effects of oral intake of water in people with dysphagia with previously identified aspiration.

## Methods

### Institution and ethics

The study was completed at a Regional Tertiary Teaching Hospital in the Gippsland region in Victoria, Australia. The application entitled "effects of thin water in patients who have been prescribed thickened fluids by a Speech Pathologist to assess hydration and quality of life", was submitted and approved by the local human research ethics committee. The study was registered with the Australian New Zealand Clinical Trials Registry (ACTRN12608000107325) and informed consent was provided by the patients or medical enduring power of attorney before inclusion.

### Participants

A total of 100 patients from the Regional Hospital in Victoria, Australia, were recruited in this study. People with dysphagia, who had been prescribed a modified or thickened fluid diet by a Speech Pathologist, over the age of 18 and without a diagnosis of chronic respiratory conditions or prior tracheostomy, were eligible for inclusion. From these, 91 completed the trial, with five being discharged before completion, two not willing to complete and two being excluded due to discomfort with ingestion of thin liquid. All patients were confirmed to aspirate thin liquids by independent assessment by two experienced speech pathologists. Aspiration of thin liquids was also verified in selected patients (10) using conventional radioactive barium videofluoroscopic swallow evaluations. Due to ethical considerations and the inherent dangers of the barium swallow test, videofluoroscopic examination is not routine for all patients exhibiting signs and symptoms of aspiration in the Regional hospital at which this study was completed. Patients undergo the procedure only when an experienced clinician is unsatisfied with the results of the assessment at the bedside examination. As mentioned, the bedside clinical examination was completed independently by two Speech Pathologists to determine the level of dysphagia and aspiration of thin liquids. Pulse oximetry and cervical auscultation were utilised as non-invasive tools to assist with the detection of silent aspiration and make a clinical judgement. We are aware of evidence regarding limitations surrounding both the pulse oximetry and cervical auscultation and where the Speech Pathologists were uncertain regarding detection of silent aspiration by clinical bedside assessment alone, a videofluoroscopy was performed. Additionally, 12 of the participants had a videofluoroscopic procedure completed within one month of being included in the study, indicating aspiration of thin liquids. Therefore, further videofluoroscopic studies were contraindicated due to the recent exposure to radiation and due to the severity dysphagia not having been altered upon bedside clinical examination. We also considered esophageal dysmotility by evaluating patient case histories, previous medical histories and current medications. Where gastroesophageal reflux was present or achalasia suspected, a barium swallow was completed (total of two participants) and the radiology reports indicated normal esophageal peristalsis.

**Figure 1:**
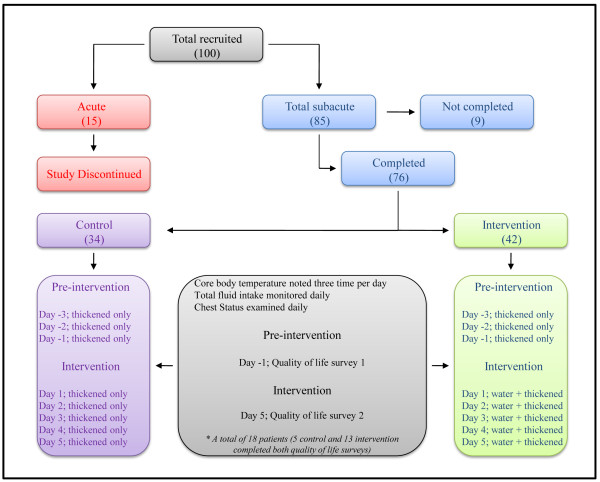
**Outline of participant groupings and study methodology**.

Initially patients were recruited from either an acute unit (total 15) or subacute units (total 85) and randomly assigned (age- and sex-matched) either to the control group (thickened fluids only; eight acute and 41 subacute) or to the intervention group (thickened fluids and free access to water; seven acute and 44 subacute). Once consent was obtained participant details were entered into a freely available database, a unique identifier was obtained and each participant was allocated into one of the study groups using the clinical trial randomizer. The research nurses and clinical speech pathologists were aware of the patient allocations however, medical staff completing the chest examinations described below, were blinded to the study. Participant groups and study interventions are outlined in Figure 1. As described in the discussion, the findings from the acute patients were not subjected to further analysis given the limitations and obvious conclusions that could be drawn from this group.

The characteristics of the subacute patients that participated in the study are summarized in Table 1. A total of 76 subacute patients completed the study and were placed in either the control (n = 34; 18 female and 16 male) or intervention (n = 42; 19 female and 23 male) groups. The mean ± standard deviation ages were 79 ± 11 and 80 ± 7 years, in the control and intervention groups, respectively. An important issue for clarification is that of underlying clinical pathology. In Table 1 the major condition diagnosed on admission by the physicians, is noted for each patient. However, patients generally suffered from multiple ailments, the most common being CVA with accompanying depression and CVA and dementia. All of the patients had been prescribed modified thickened consistencies by qualified Speech Pathologists as indicated (Table 1).

**Table 1:** Control and intervention group subacute patient characteristics

	Control	Intervention
**Patients**		
Total	34	42
Age (mean ± SD)	79 ± 11	80 ± 7
Female	18	19
Male	16	23

Pathology*		
CVA**	15	25
Dementia	6	5
Alzheimer's disease	3	4
Parkinson's disease	1	4
Cancer	6	4
Motor neuron disease	1	0
Huntington's disease	1	0
Motor vehicle accident	1	0

**Oral**		
Own teeth	7	8
Dentures	25	31
No teeth	2	3

**Mobility**		
Mobile	13	26
Low mobility	13	12
Immobile	8	4

**Modified Diet**		
Puree/Honey	18	17
Puree/Pudding	3	2
Puree/Nectar	1	3
Minced/Honey	6	10
Minced/Nectar	2	3
Soft/Minced/Honey	1	3
Soft/Minced/Nectar	3	4

### Study Design

The study was completed in two distinct stages. In the first, education sessions were provided to all nursing, medical and allied health staff allocated to areas in which people with dysphagia were to be admitted. Furthermore, an organisation wide oral hygiene protocol was implemented. Nursing staff were given specific training in the implementation or promotion of vigorous oral hygiene as well as strict parameters for the provision of water to participants in the study. Water could only be provided, at least 30 minutes after a meal, following thorough oral toilet which was identical for all patients across the control and intervention groups. The oral hygiene screening tool developed for nursing staff and utilised for all patients involved a thorough brushing of teeth or cleaning of dentures, to ensure there was no food build up or residue noted on or between teeth. Chlorhexidine mouthwash was used where necessary to ensure thorough cleaning of the oral cavity prior to provision of water.

In the implementation phase (stage 2), the recruited patients were monitored for 72 hours while they consumed only the prescribed thickened fluids and modified consistencies. Following this initial pre-intervention phase (referred to as Days -3 to -1 throughout this paper), participants in the intervention group consumed the prescribed thickened modified diet and under strict nursing guidance, were given access to water as requested. Participants in the control group continued to consume the prescribed thickened diets only. All participants were monitored for a further five days (referred to as the post-intervention phase, Days 1 to 5 throughout this paper).

### Data collection

Patients were examined for chest status daily by experienced physicians (blinded to patient assignment) and any irregularities were noted in the patient medical file. After careful consultation with four medical experts, a period of five days for the determination of aspiration pneumonia was recommended. The principle being that pneumonia would most likely develop within 24-48 hours of aspiration of thin liquids. Although we are aware that aspiration pneumonia may occur beyond the five day period, the opinion of experts was considered and adopted for the study. Further, nursing staff recorded core body temperature three times per day for the entire eight day period. In addition, total daily fluid intake for each participant was noted daily.

### Quality of life surveys

Quality of life surveys were administered on the final day of the pre-intervention period (Day -1) and on the final day of the post-intervention period (Day 5). A total of 18 participants, 5 from the control group and 13 from the intervention group, completed the survey. Patients were asked the following series of four simple questions related to quality of life:

1. How have you been feeling?

2. Are you happy with the drinks?

3. Have you been feeling thirsty?

4. How clean does your mouth feel?

Participants were asked to point to one of six faces that best represented their feeling and were assessed on the basis of the Wong and Baker, 1997, pain scale rating chart [30]. This test assesses pain on a zero to ten scale with an increment of two, with the use of drawn faces ranging from a smiley to a crying sad face, and was adapted to be utilised for the purpose of defining quality of life [30].

### Statistical analysis

Paired t-tests were performed to calculate p-values for evaluating statistical significance for the hydration and quality of life surveys. The low response rate (overall 24%) for the quality of life surveys is likely to bias the data particularly given that only 5 from the control group and 13 from intervention group completed the surveys. However, it is noteworthy that the dissatisfaction of people with dysphagia to thickened diets is well known and non-compliance is a very common clinical problem.

## Results

### Lung related complications

**Figure 2:**
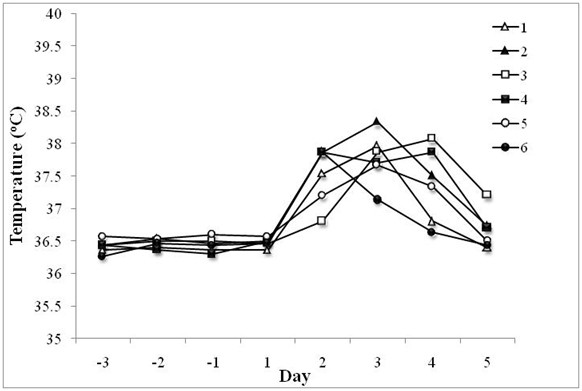
**Core body temperature changes in patients that were diagnosed with aspiration pneumonia or bibasal crepitations**. The core body temperature of each patient on the study was monitored three times per day for the three day (Days -3 to -1) pre-intervention period and five days (Days 1 to 5) post-intervention. The mean daily temperature of the six patients in the intervention group (each patient is indicated by a different symbol), that developed aspiration pneumonia or bibasal crepitations is shown. The findings indicate a spike in mean core body temperature two to three days after the ingestion of water, corresponding to the time of the first signs of aspiration pneumonia or bibasal crepitations. In all cases, the rise in temperature subsided with the administration of antibiotics.

Our findings indicate that six patients (14.3%; two female and four male) developed lung related complications in the intervention group, with three (7.1%) being diagnosed with aspiration pneumonia and three (7.1%) had lower quadrant bibasal crepitations (indicative of aspiration pneumonia but not confirmed) [31]. As expected an increase in mean core body temperature, corresponding to the time of diagnosis of the first signs of respiratory symptoms by experienced physicians, was noted in these patients (Figure 2). The increase in core body temperature and the first signs of aspiration pneumonia or bibasal crepitations typically occurred two to three days after the ingestion of water. In contrast, to the intervention group, none of the patients in the control group developed lung related problems during the study.

### Daily fluid intake

**Figure 3:**
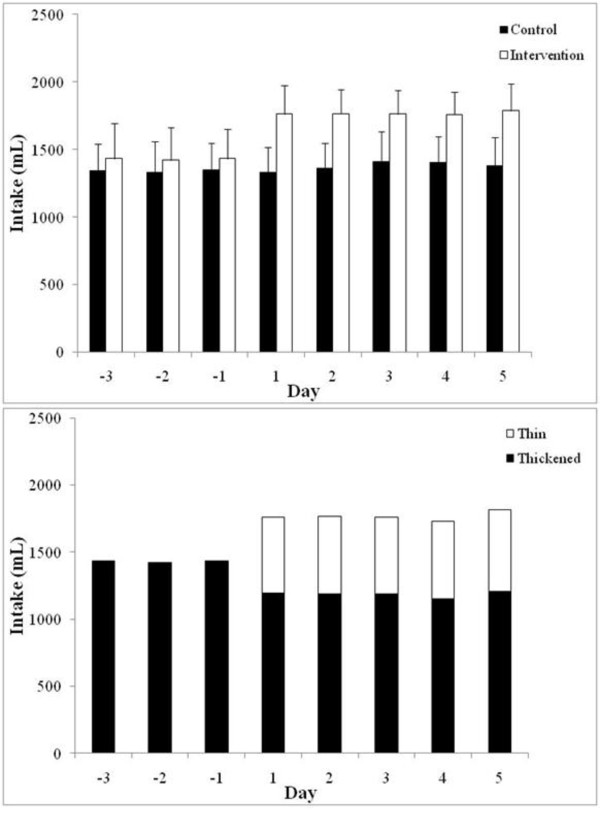
**Total daily oral liquid intake by the patients in the study**. Comparison of the mean total daily oral liquid intake by patients in the control and intervention groups (A). Patients in the control group were allowed only thickened fluids for the total eight day period (Days -3 to -1 pre-intervention and Days 1 to 5 post-intervention). Patients in the intervention group were allowed access to water for the five day post-intervention period. The findings indicate a significant difference in the total fluid intake in the intervention group compared to the control group after the allowance of water (Days 1 to 5). Comparison of the mean proportions of daily intake of thickened and thin (water) fluids in the intervention group (B). The findings indicate only a modest decrease in the volume of daily intake of thickened fluids and an overall increase in the total fluid intake, following the allowance of water in the intervention group (Days -3 to -1 compared to Days 1 to 5).

Patients in the control group were allowed only thickened fluids for the total eight day period (Days -3 to -1 pre-intervention and Days 1 to 5 post-intervention), and patients in the intervention group were allowed access to water for the five day post-intervention period. The total daily oral liquid intake by each of the participant in the study was noted. The findings indicate that the two groups had a similar intake of thickened fluids during the three day (Days -3 to -1) pre-intervention period with a mean ± standard deviation of 1340 ± 9.5 mL and 1428 ± 7.0 mL for the control and intervention cohorts, respectively. Comparison of the mean total daily oral liquid intake after the allowance of water (Days 1 to 5) for the intervention group highlights a significant difference with a mean ± standard deviation of 1378 ± 33.7 mL and 1767 ± 10.7 mL (P = <0.001), for the control and intervention groups, respectively (Figure 3A). The increase in total fluid intake in the intervention group, from a daily mean of 1428 mL in the pre-intervention period to 1767 mL (P = <0.001) during the intervention period, represents a modest decrease in thickened fluids to a mean ± standard deviation of 1185 ± 20.7 mL. However, adequate compensation in terms of hydration was provided with the supplementation of water which accounted for a daily mean ± standard deviation of 582 ± 15.8 mL during the post-intervention period (Figure 3B). It should be noted that two patients from the control group and one patient from the rehabilitation group required intravenous fluid during the study.

### Quality of life

**Figure 4:**
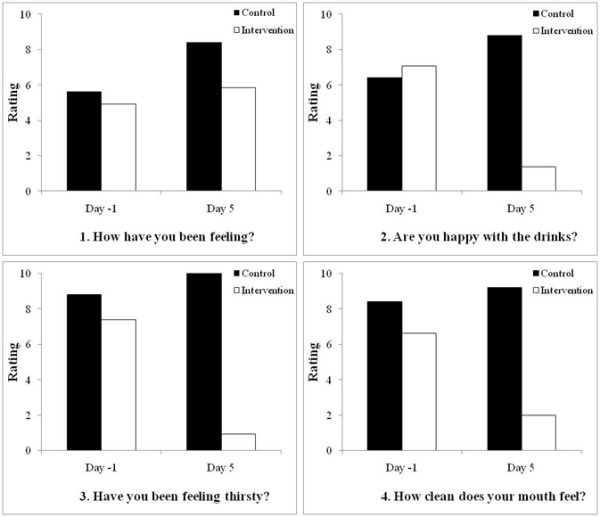
**Results of the quality of life surveys for patients in the control and intervention groups**. Patients were asked a series of four simple questions related to quality of life on the final day of the pre-intervention period (Day -1) and on the final day of the post-intervention period (Day 5). Scores represent the mean of each group on the basis of the Wong and Baker, 1986, faces rating chart which assesses pain on a zero to ten scale with an increment of two; therefore, the lower scores represent lower pain or greater happiness (0 is no pain, represented by a smiley face and 10 is worst pain imaginable, represented by sad weeping face). It is interesting to note the remarkable differences in the two groups when asked a specific question (questions two to four), with the intervention group showing much greater satisfaction than the control group. In contrast, the difference is much smaller between the two groups, with the control group indicating slightly more positive responses than the intervention group, for the more general question one.

Quality of life surveys were administered on the final day of the pre-intervention period (Day -1) and on the final day of the post-intervention period (Day 5). The survey comprised of four questions related to the feelings of the participants and the Wong and Baker, 1986, faces rating chart was used to calculate scores. The findings indicate that the patients in both the control and intervention groups were generally feeling okay (mean scores 5.6 and 4.9, respectively) at the end of the pre-intervention period in which all patient were on a thickened modified diet for three days (Figure 4). However, during the same period they were largely dissatisfied when asked about how they felt about the drinks (mean 6.4 and 7.1 for the control and intervention groups, respectively), their level of thirst (mean 8.8 and 7.4) and mouth cleanliness (mean 8.4 and 6.6). Profound differences were observed between the two groups in the follow-up survey at the end of the study. The intervention group reported remarkably higher levels of satisfaction with the drinks (mean 8.8 and 1.4 for the control and intervention groups, respectively; P = <0.001), their level of thirst (mean 10 and 0.9; P = <0.001) and mouth cleanliness (9.2 and 2.0; P = <0.001) than the control group. Intriguingly, this did not appear to correspond to an overall increase in positive feeling. Although the intervention group did report a more positive response than the control group (means 5.8 and 8.4, respectively; P = 0.094), there appeared to be a slightly worse general feeling amongst the group compared to the pre-intervention period (means for the intervention group were 4.9 and 5.8 at the end of the pre-intervention and post-intervention periods, respectively; P = 0.111).

## Discussion

Our findings indicate that six subacute patients (14.3%) in the intervention group developed lung related complications during the study. The increase in core body temperature corresponding with the diagnosis of lung crepitations typically occurred two to three days after the ingestion of water, indicating that close monitoring of patients is critical during the initial 72 hour period from the deviation from the thickened fluid or modified diet.

The significant difference in patients in the intervention (14.3%) compared to the control group (no cases), suggests a causal relationship between aspiration of water and the development of lung complications including aspiration pneumonia. This is in contrast with the findings from a previously published authoritative paper, in which it was found that none of the stroke patients with identified aspiration of thin liquids developed lung complications as a result of being given access to water [29]. The major differences between the two studies are the number of patients (ten per group in the previous study compared to 42 in the intervention group in this study) and the selection criteria. In the previous only newly admitted CVA patients were included and patients with previous CVA, neurodegenerative diseases, multiple medical diagnoses and those unable to self-feed were excluded from the study [29]. In contrast the majority of our participants had multiple medical diagnoses including disease other than CVA and patients with degenerative neurologic dysfunction and immobile patients were included.

In our study, of the six participants (two female and four male) that developed lung complications in the intervention group, two had Alzheimer's disease, two had Parkinson's disease, one had a congenital intellectual disability and the other suffered from cancer. One of the Parkinson's disease and the cancer patients also had a CVA. Another feature of the patients that developed lung complications was that three were classified as being immobile and three had low mobility; immobile patients were bedridden and required assistance for feeding and patients with low mobility were predominantly bedridden but able to self-feed and walk with limited assistance. Given that patients with similar characteristics in the intervention group (seven) did not develop lung complications, suggests that neurological dysfunction and immobility or low mobility can be considered as increased risk factors but not a certainty for the development of aspiration-induced lung complications. Importantly, had we considered only the newly admitted CVA patients with relative mobility (total of 12) in the intervention group, our findings would indicate no cases of lung complications and be in complete accordance with the previously published findings [29].

One of the well known issues with the management of people with dysphagia is the discontent and in numerous cases the refusal to adhere to diets composed of only thickened fluids and modified solid consistencies [27,28]. Apart from the ethical considerations, which are important and discussed below, this may represent serious issues with hydration. This was highlighted in our findings of daily fluid intake which indicate that patients in the intervention group consumed an average of >300 mL more fluid after water was allowed compared to their intake in the pre-intervention period and compared to the fluid intake of the control group. It was noted previously, and quite surprisingly, that the access to water did not result in a dramatic decrease in the intake of thickened fluids and the intake of water did not exceed that of thickened fluids for any of the participants [29]. This is in accordance with our findings which indicate the mean intake of thickened fluids for the intervention group during the post-intervention period was 1185 ± 21 mL and mean water intake was 582 ± 16 mL. Similarly, the amount of water consumption did not exceed thickened fluid intake for any of the patients. Regarding hydration, only one patient required intravenous fluid in the intervention group, and this was during the pre-intervention phase when access to water was not allowed. Two patients in the control group required intravenous fluid.

Only five (15%) and 13 (31%) patients from the control and intervention groups, respectively, completed the quality of life surveys in our study. The main reasons for this low completion rate are cognitive inability which accounts for many of the participants and also we were not persistent with patients who were experiencing difficulties on any of the two assessment days. Although this low completion rate may bias the results, the overall findings of the quality of life surveys in combination with our knowledge from extensive (over 10 years) clinical experience, indicate the dissatisfaction of patients to diets composed of only thickened fluids and modified solid consistencies. Remarkable differences were observed between the two groups in the follow-up survey at the end of the study with the intervention group reporting much higher levels of satisfaction with the drinks, their level of thirst and mouth cleanliness compared to the control group. As mentioned earlier, it is intriguing, that this did not appear to correspond to an overall increase in positive feeling. Admittedly, this was most likely due to a poor choice of generic question (How are you feeling?) which is, with hindsight, inappropriate for the cohort of participants.

Finally, it is important to note that we initially designed the study in such a way as to collect data for an acute-phase cohort. However, the acute group represented unique problems related to data reliability, with the patients included in the intervention cohort refusing thickened consistencies and the majority requiring intravenous fluids. Although the acute patients were initially included in the study and we attempted to monitor them according to the protocol, serious complications including the development of aspiration pneumonia in the intervention group as a result of feeding at risk (i.e. they refused thickened fluids and modified solid consistencies) prompted us to re-think the strategy. Although ethical considerations related to quality of life are important, our observations highlight the need to strongly encourage thickened fluids and modified solid consistencies to acute patients. Given the complications with this group and the obvious recommendation, the data did not require analysis, no further consideration of this cohort is appropriate.

## Conclusions

Overall, our findings indicate a significantly increased risk in the development lung complications in patients given access to water when they are known to aspirate thin liquids. However, we appear to have narrowed down patients at highest risk, namely those with degenerative neurologic dysfunction who are immobile or have low mobility. On the basis of our findings we recommend that acute patients, patients with severe neurological dysfunction and immobility should be strongly encouraged to adhere to a thickened fluid or modified solid consistency diet. In the case of rehabilitation patients, a previous authoritative study concluded that access to water should be permitted when there is a refusal to consume thickened fluids or modified solid consistencies and when hydration becomes a medical problem. On the basis of our findings, particularly, our daily fluid intake and survey data, we propose an extension to this conclusion by recognising that the ethical considerations are too strong to ignore. We recommend subacute patients with relatively good mobility should have choice after being well-informed of the relative risk. Indeed, our current findings do not obviate the need to deviate from the Frazier Rehab Center free water protocol.

Research aimed at further identification of subsets of patients with a high risk of developing aspiration pneumonia is warranted. In addition, clarification of risk factors for lung complications following the ingestion thin liquids, including water, in people with dysphagia is important. Perhaps, further consideration should be given to factors such as bacterial colonisation and immunological competence.

## Competing interests

The authors declare that they have no competing interests.

## Authors' contributions

MJP conceived of the study, and participated in its design, coordination and data collection. LC was involved in the study design and data collection. TCK was involved in study design and preparation of the manuscript. All authors read and approved the final manuscript.

## Pre-publication history

The pre-publication history for this paper can be accessed here:

http://www.biomedcentral.com/1471-2318/11/9/prepub
